# Unveiling divergent treatment prognoses in IDHwt-GBM subtypes through multiomics clustering: a swift dual MRI-mRNA model for precise subtype prediction

**DOI:** 10.1186/s12967-024-05401-6

**Published:** 2024-06-18

**Authors:** Qiang Ji, Yi Zheng, Lili Zhou, Feng Chen, Wenbin Li

**Affiliations:** 1 Department of Neuro-Oncology, Cancer Center, China National Clinical Research Center for Neurological Diseases, Beijing Tiantan Hospital, Capital Medical University, Beijing, China; 2 China National Clinical Research Center for Neurological Diseases, Beijing, China; 3 National Institute for Data Science in Health and Medicine, Capital Medical University, Beijing, China

**Keywords:** Bioinformatics, Radiomics, Glioblastoma, IDH wild type, Multiomics, Machine learning, Programmed cell death, Online interactive platform

## Abstract

**Background:**

IDH1-wildtype glioblastoma multiforme (IDHwt-GBM) is a highly heterogeneous and aggressive brain tumour characterised by a dismal prognosis and significant challenges in accurately predicting patient outcomes. To address these issues and personalise treatment approaches, we aimed to develop and validate robust multiomics molecular subtypes of IDHwt-GBM. Through this, we sought to uncover the distinct molecular signatures underlying these subtypes, paving the way for improved diagnosis and targeted therapy for this challenging disease.

**Methods:**

To identify stable molecular subtypes among 184 IDHwt-GBM patients from TCGA, we used the consensus clustering method to consolidate the results from ten advanced multiomics clustering approaches based on mRNA, lncRNA, and mutation data. We developed subtype prediction models using the PAM and machine learning algorithms based on mRNA and MRI data for enhanced clinical utility. These models were validated in five independent datasets, and an online interactive system was created. We conducted a comprehensive assessment of the clinical impact, drug treatment response, and molecular associations of the IDHwt-GBM subtypes.

**Results:**

In the TCGA cohort, two molecular subtypes, class 1 and class 2, were identified through multiomics clustering of IDHwt-GBM patients. There was a significant difference in survival between Class 1 and Class 2 patients, with a hazard ratio (HR) of 1.68 [1.15–2.47]. This difference was validated in other datasets (CGGA: HR = 1.75[1.04, 2.94]; CPTAC: HR = 1.79[1.09–2.91]; GALSS: HR = 1.66[1.09–2.54]; UCSF: HR = 1.33[1.00–1.77]; UPENN HR = 1.29[1.04–1.58]). Additionally, class 2 was more sensitive to treatment with radiotherapy combined with temozolomide, and this sensitivity was validated in the GLASS cohort. Correspondingly, class 2 and class 1 exhibited significant differences in mutation patterns, enriched pathways, programmed cell death (PCD), and the tumour immune microenvironment. Class 2 had more mutation signatures associated with defective DNA mismatch repair (P = 0.0021). Enriched pathways of differentially expressed genes in class 1 and class 2 (P-adjust < 0.05) were mainly related to ferroptosis, the PD-1 checkpoint pathway, the JAK-STAT signalling pathway, and other programmed cell death and immune-related pathways. The different cell death modes and immune microenvironments were validated across multiple datasets. Finally, our developed survival prediction model, which integrates molecular subtypes, age, and sex, demonstrated clinical benefits based on the decision curve in the test set. We deployed the molecular subtyping prediction model and survival prediction model online, allowing interactive use and facilitating user convenience.

**Conclusions:**

Molecular subtypes were identified and verified through multiomics clustering in IDHwt-GBM patients. These subtypes are linked to specific mutation patterns, the immune microenvironment, prognoses, and treatment responses.

**Supplementary Information:**

The online version contains supplementary material available at 10.1186/s12967-024-05401-6.

## Introduction

Glioblastoma (GBM) is the most lethal primary brain tumour, with a median survival duration of only 15 months and a mere 5% of patients surviving after five years [1, 2]. Its aggressive infiltration status, diverse genetic makeup, and formidable shield, the blood–brain barrier (BBB), make treatment efforts challenging [3]. GBM's formidable defences make it a tragic pinnacle in the broader cancer landscape, where resistance and relapse are unfortunately not uncommon.

Before the 2021 WHO classification standards for the central nervous system (CNS), the classification of glioblastoma (GBM) relied solely on histology [4]. However, with the advent of molecular detection technologies and large-scale cancer population gene sequencing, our understanding of GBM has significantly improved [5, 6]. We progressed from initially recognising the impact of single-gene chromosomal molecular changes, such as those to IDH, MGMT, and 1p/19q [7, 8], on GBM treatment and prognosis. Subsequently, we determined that comprehensive patterns of changes, such as EGFR amplification, a combination of whole chromosome 7 gain and whole chromosome 10 loss (7 + /10−), or TERT promoter (pTERT) mutations, lead pathologically diagnosed low-grade gliomas to exhibit adverse outcomes similar to those of GBM [9].

The 2021 WHO CNS classification standards have introduced comprehensive GBM standards that account for molecular and pathological factors[10]. The diagnostic criteria included patients with a histological diagnosis of glioblastoma (histGBM) and IDH wild-type status. Diffuse astrocytic tumours lacking the histological features of glioblastoma are designated molecular GBM (molGBM, WHO grade 4) if they exhibit specific molecular abnormalities, such as TERT promoter mutation, EGFR amplification, or chromosomal + 7/− 10 copy changes. The 2021 WHO classification standards for the central nervous system (CNS) have generated some controversy regarding the classification of GBM due to inconsistent research findings on the differences in survival between histGBM and molGBM patients [11]. Tesileanu et al. [12] and Berzero et al. [13] presented opposing views: the former argue that the prognoses for histGBM and molGBM are similar, while the latter contend that the prognoses for histGBM and molGBM are different. This may be because the classification of molGBM only considers the mutation patterns of a few molecules, thus having certain limitations. Therefore, a thorough analysis of multiomics data from patients can enhance our understanding of the regulatory mechanisms specific to certain diseases [14, 15]. Unfortunately, current research on the classification of IDHwt-GBM has focused primarily on single-dimensional studies, with few involving multiomics approaches [16–18]. Furthermore, there has been a lack of practical tools generated from these studies for easy use. Our research aims to address these issues.

In this study, we utilised 10 multiomics integration strategies to develop a comprehensive consensus subtype for IDHwt-GBM patients following the 2021 WHO CNS classification standards. By analysing the relationships among IDHwt-GBM subtypes, molGBM subtypes, and histGBM subtypes, we aimed to elucidate the conflicting research findings on survival differences. Our findings revealed the significant prognostic value of IDHwt-GBM subtypes and their role in predicting responses to radiotherapy and temozolomide combination therapy. Additionally, to facilitate clinical use, we created predictive models based on mRNA and MRI data, along with an online interactive platform at https://xqqcc.shinyapps.io/IDH1wtGBM/.

## Materials and methods

### Data collection and study population

Primary cases of IDH1wt-GBM were identified from six glioma cohorts by extracting data that met the WHO 2021 classification criteria [10]. The following cohorts were used in the current study: The Cancer Genome Atlas (TCGA), the Chinese Glioma Genome Atlas (CGGA_325), Glioma Longitudinal AnalySiS (GLASS), The National Cancer Institute's Clinical Proteomic Tumor Analysis Consortium (CPTAC), the University of Pennsylvania Glioblastoma (UPenn), and the University of California San Francisco Preoperative Diffuse Glioma MRI (UCSF-PDGM) cohorts. As the patients in TCGA possess multidimensional data, including genomic, radiomic, and treatment information, all exploratory findings in this study are based on the TCGA cohort. Other independent cohorts were used to validate and confirm the discoveries made in TCGA. Specifically, GLASS is employed to validate treatment-related findings, while CGGA_325, GLASS, and CPTAC are used to validate genomics-related discoveries. UPenn and UCSF-PDGM were used to validate the findings at the radiomic level. Detailed database information is described in the Supplementary Datasets and Table S1.

### Multiomics consensus ensemble analysis

This study represents a comprehensive multiomics analysis in which ten sophisticated clustering algorithms were used to identify hidden patient groupings within a cohort of 184 individuals. We examined the intricate relationships between mRNA, lncRNA, and gene mutation data by employing a diverse array of methods that have been rigorously evaluated in the literature [19]. To transcend individual algorithm biases and paint a more robust landscape, we embraced the power of consensus clustering. This meticulous approach blends the insights gleaned from each method, culminating in a powerful classification scheme. To ascertain the optimal number of clusters, we employed the guiding principles of the Gap statistics [20]. The MOVICS package [21] was our optimised tool, with functions such as getConsensusMOIC and getMOIC seamlessly integrating clustering and consensus building. Detailed information on the multiomics consensus is described in Supplementary Methods 1.

### Subtype prediction employing a model-free approach based on mRNA data

To make the subtypes identified in TCGA widely applicable to other datasets and the real world, considering that mRNA is the most detected data type in patients, we used the differentially expressed genes identified by multiomics clustering in TCGA to predict subtypes using partition around medoids (PAM). Specifically, we calculated the Pearson correlation coefficient between each patient and the clustering centres of different subtypes in TCGA. Then, each sample in the validation cohort was assigned to the subtype label with the highest Pearson correlation coefficient [22].

### Bioinformatics analysis of differences between subtypes of IDH1-wtGBM

We assessed differences in gene expression, somatic mutations, mutational signatures, gene enrichment pathways, the immune microenvironment, and programmed cell death (PCD) pathways between IDH1wt-GBM subtypes. Please refer to Supplementary Methods 2 for detailed information.

### Screening of potential therapeutic drugs

In this study, we utilised the Gene Set Cancer Analysis (GSCA) [23] online drug analysis tool to identify relevant drugs by analysing the differentially expressed genes between IDH1-wtGBM subtypes. Additionally, we used the R package OncoPredict [24] to predict potential drug responses, specifically half-maximal inhibitory concentration (IC50) values, across different IDH1wt-GBM subtypes.

### Establishment of a machine learning-driven model for predicting subtypes of IDH1-wtGBM through radiomic features

To increase the applicability of molecular subtyping in IDH1wt-GBM patients and provide noninvasive options for patients who are ineligible for biopsy and sequencing, we extracted radiomic features from four common functional magnetic resonance imaging sequences (T1, T1C, T2, and FLAIR) from 42 patients in the TCGA dataset. We built a random forest model to predict molecular subtypes. The hyperparameters of the random forest were determined using a random grid search combined with the maximum mean accuracy in fivefold cross-validation. After training the model on TCGA data, we applied it to two independent radiomic cohorts to identify IDH1wt-GBM subtypes. The specific details of radiomic feature extraction are described in Supplementary Methods 3.

### Multifactor survival analysis and survival model validation

COX analysis was conducted on the training set and five additional independent datasets to assess the hazard ratios for various risk factors, including the IDH1wt-GBM subtypes. A meta-analysis was then performed to obtain more robust conclusions. Finally, a multifactor survival prediction model was established on the training set and validated on the test set to assess its accuracy and clinical utility. For specific details, please refer to Supplementary Method 4.

### Statistical analysis

The reported statistical significance level was two-sided, and the threshold was set at 0.05. Differences in clinical characteristics between the test and training sets were tested using the t-test, chi-square test, and nonparametric test. Multiple comparisons were corrected for P values using the Benjamini and Hochberg method.

## Results

### Study design and patient characteristics

**Fig. 1:**
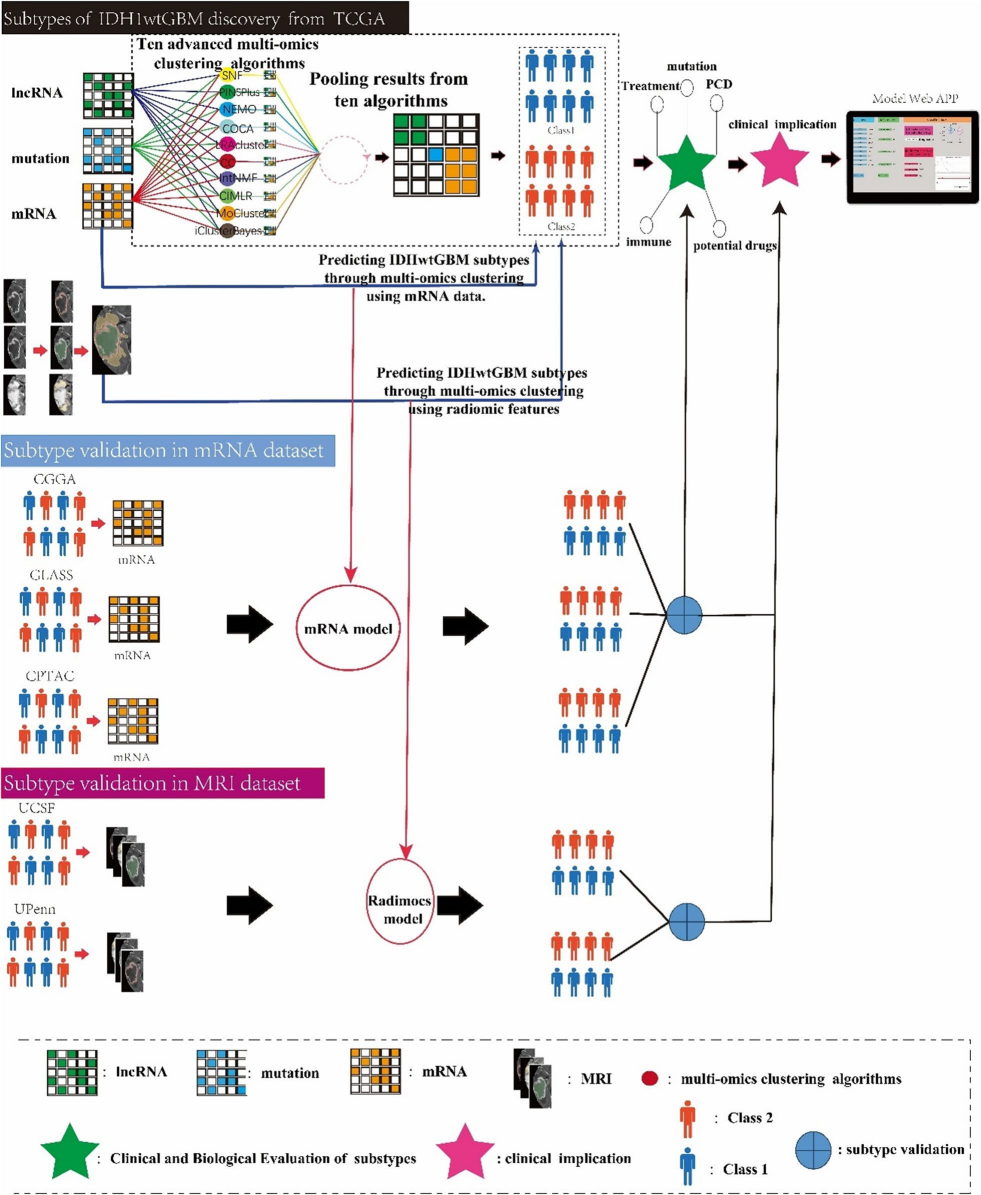
Graphical illustration of our study

Four retrospective cohorts with genomic data for IDH1wt-GBM patients were used to assess and independently validate the prognostic and treatment implications of molecular subtypes identified through multiomics data clustering within the TCGA cohorts. Furthermore, three retrospective cohorts with MRI data of IDH1wt-GBM patients were utilised to construct and validate these molecular subtypes (Fig. 1). As shown in Table S1, the discovery set consisted of 184 IDH1wt-GBM patients from the TCGA cohort. Of these patients, 114 (62.0%) were male and 70 (38.0%) were female, with a median age of 60 years (IQR: 53.0–67.0 years). The CGGA-325 cohort included 74 IDH1wt-GBM patients, of which 48 (64.9%) were male and 26 (35.1%) were female. The median age of this cohort was 53.5 years (IQR: 44–58.75 years). The GLASS cohort comprised 103 patients with IDH1wt-GBM, of which 66 (64.1%) were male and 37 (35.9%) were female, with a median age of 54 years (IQR 46.0–63.5 years). The CPTAC cohort consisted of 92 patients with IDH1wt-GBM, of which 51 (55.4%) were male and 41 (44.6%) were female, with a median age of 59.0 years (IQR 51.0–67.0 years). The UPenn cohort consisted of 374 patients with IDH1-wtGBM, 228 (61.0%) of whom were male and 146 (39.0%) of whom were female, with a median age of 64.3 years (IQR: 52.3–72.0 years). The UCSF-PDGM cohort comprised 360 patients with IDH1wtGBM, of whom 215 (59.7%) were male and 145 (40.3%) were female, with a median age of 62 years (IQR: 54.8–70.0 years).

### Multiomics consensus molecular subtypes of IDH1-wtGBM

**Fig. 2:**
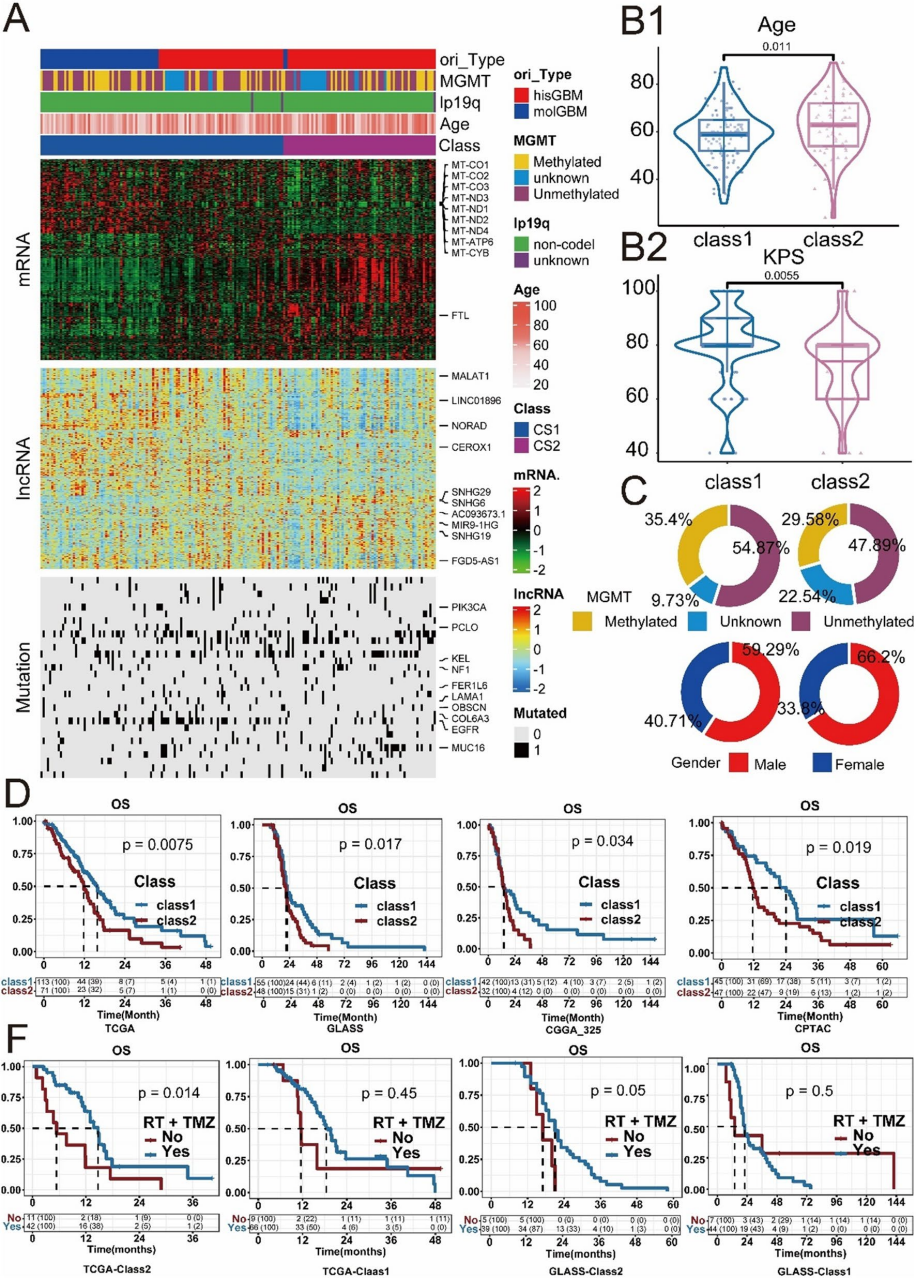
The multiomics integrative consensus subtypes of IDH1wt-GBM. **A** Comprehensive heatmap of consensus ensemble subtypes, including mRNA, lncRNA and mutant gene. **B**–**C** Population Characteristics of the New IDH1wt-GBM Subtype. **B1** Age Difference in the IDH1wt-GBM Subtype. **B2** Karnofsky Performance Status (KPS) in the IDH1wt-GBM Subtype. **C** O-6-methylguanine-DNA methyltransferase (MGMT) Status and Gender in the IDH1wt-GBM Subtype. **D** Survival analysis of IDH1wtGBM patients with class1 and class2 in the TCGA (development cohort), GLASS, CGGA-325 and CPTAC (validation cohort). **F** Different survival outcomes among the combination of radiotherapy and temozolomide. RT + TMZ: combination of radiotherapy and temozolomide

As shown in Fig. 1, we identified two subtypes using 10 multiomics ensemble clustering algorithms. The number of subtypes was determined by comprehensively referring to the gap statistical analysis (Figure S1). The clustering results were combined using the consensus ensemble approach, which revealed distinctive molecular expression patterns across mRNAs, lncRNAs, and somatic mutations (Fig. 2A and Figure S2). The classification system closely correlated with overall survival (OS) (p = 0.007; Fig. 2D). Subtype 1 (class 1) had the most favourable survival advantage and was more prevalent in younger and higher Karnofsky Performance Status (KPS) populations (refer to Fig. 2B). The results of the differential expression analysis among subtypes were analysed. We selected the top 9 genes with the greatest fold changes (Table S2). The partition around medoids (PAM) method was used to predict the subtype of patients in the external validation cohort. In essence, each sample in the validation cohort was assigned a subtype label based on the centroid with the highest Pearson correlation with the sample [22]. Subsequently, the classifiers were validated in multiple external cohorts to further validate the stability of subtypes. As shown in Fig. 2D, consistent with the TCGA cohort, patients in class 1 had a better prognosis in the three external validation cohorts (GLASS, CGGA-325, and CPTAC). Notably, in the TCGA cohort, individuals in class 2 appeared to be more responsive to the combination of radiotherapy and temozolomide (Fig. 2F, P = 0.014), whereas no such difference was observed in class 1 (Fig. 2F, P = 0.45). Furthermore, a separate GLASS cohort that provided information on drug therapy also confirmed this trend of treatment disparities (Fig. 2F class 2, P = 0.05; class 1, P = 0.5). These findings from the TCGA and GLASS cohorts could provide valuable insights into therapeutic approaches for gliomas.

### Somatic mutations and mutational signatures related to the molecular subtypes of IDH1-wtGBM.

**Fig. 3:**
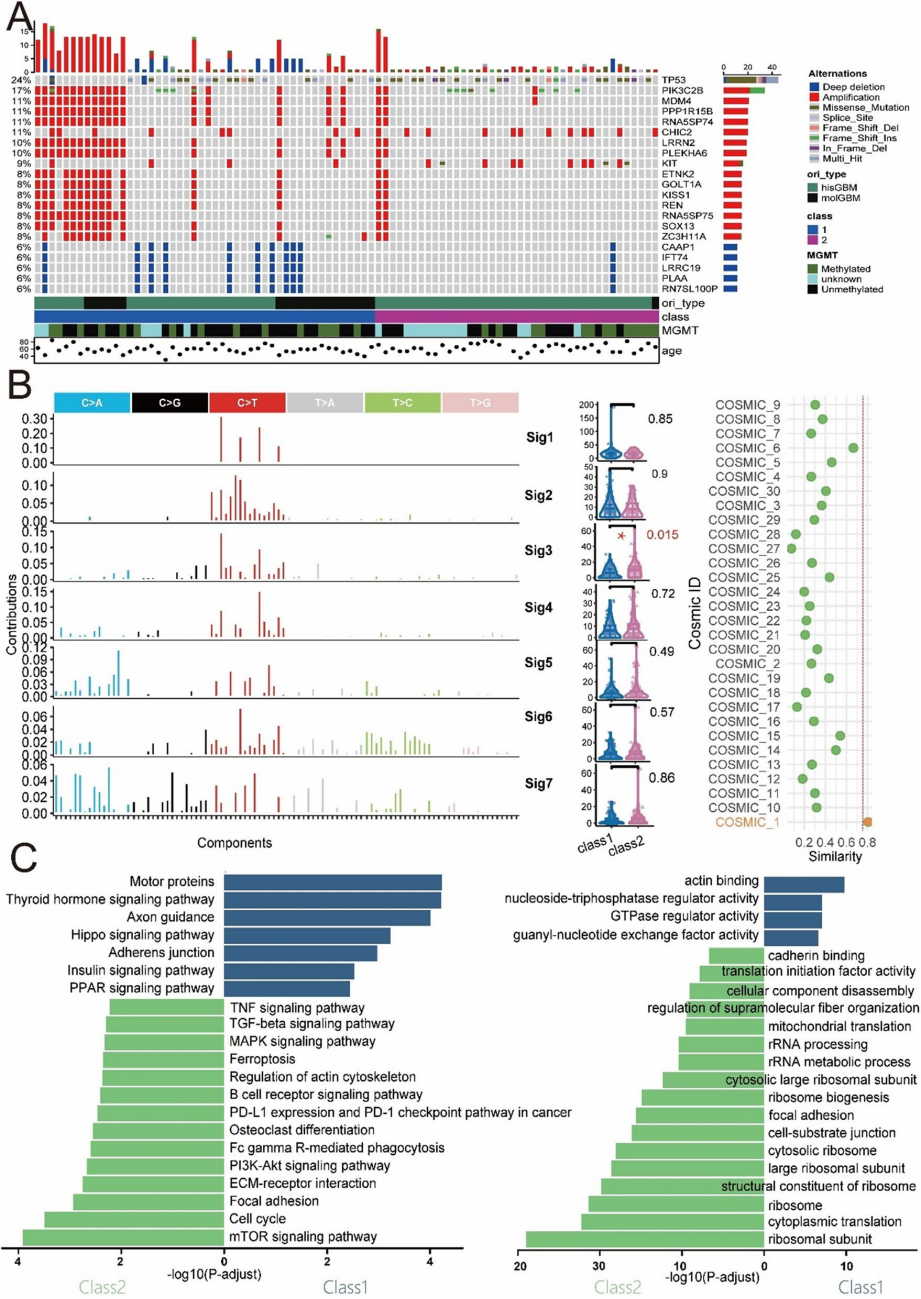
Somatic mutations, mutational signatures and pathway enrichment analysis. **A** Mutational landscapes of class 1 and class 2 show differences in genetic mutations. **B** Mutational spectrum of the seven de novo mutational signatures extracted by the Sigflow analysis (left);Differential Analysis of Mutational Signatures Between Class 1 and Class 2 (middle); Matching Sig3 with COSMIC Reference Signatures(right). **C** KEGG and GO pathway enrichment analysis. The bars extending to the right indicate activation in class 1, while those extending to the left indicate activation in class 2

Figure 3A displays the mutated genes with an overall mutation frequency greater than 6% and differences among subtypes (P < 0.05) based on the results of the differential somatic mutation analysis among subtypes (Table S3). TP53 mutations were more prevalent in class 2, patients with poorer survival, consistent with previous research conclusions [25]. Furthermore, it is worth noting that the CHIC2 and KIT genes, which are located on chromosomal segment 4q12, had a greater amplification frequency in Class 2. This finding is consistent with the conventional understanding that amplification at 4q12 is associated with a poorer prognosis [26]. The analysis of mutational signatures can reveal the molecular mechanisms that influence the somatic mutations observed in cancer genomes. As shown in Fig. 3B, mutational analysis using BayesNMF [27] identified seven new signatures. The third signature, sig3, exhibited greater absolute exposure in class 2 (P = 0.015). Matching sig3 to the COSMIC reference signatures revealed the highest cosine similarity with COSMIC_1 (SBS1), followed by SBS6 and SBS15. SBS1 is produced by the deamination of 5-methylcytosine to thymine, resulting in G:T mismatches in double-stranded DNA and subsequent C-to-T substitutions [28]. Previous research has also suggested an association between SBS1 and temozolomide treatment in glioblastomas. Individuals with recurrent glioblastomas after temozolomide therapy exhibit lower levels of SBS1 [29].

### Molecular annotation underlying molecular subtypes of IDH1-wtGBM

To understand the molecular differences between subtypes, we used transcriptome data from the TCGA cohort. After performing differential expression analysis between subtypes using the edgeR (version. 3.38.4) [30] and DESeq2(version 1.36.0) [31] methods (Table S4), we identified differentially expressed genes (DEGs) based on statistical significance criteria (P-adjust < 0.05, |log2FC|> 1). We then ranked the DEGs in descending order according to their log2-fold change. This ranked list was used for gene set enrichment analysis (GSEA). The results showed significant enrichment in pathways such as the IL-17 signalling pathway, JAK-STAT signalling pathway, chemokine signalling pathway, PI3K-Akt signalling pathway, and cytokine‒cytokine receptor interaction, among others (Table S5, Figure S3, all with P-adjust < 0.05). KEGG analysis revealed that genes significantly enriched in PD-L1 expression, the PD-1 checkpoint pathway, ferroptosis, the TNF signalling pathway, the PI3K-Akt signalling pathway, the mTOR signalling pathway, and the B-cell receptor signalling pathway were significantly enriched (Fig. 3C, Table S6). These pathways are primarily associated with programmed cell death (PCD) and environmental information processing. Gene Ontology (GO) functional enrichment analysis revealed significant enrichment (FDR < 0.05) for several categories, including cytoplasmic translation, mitochondrial translation, cellular component disassembly, cell-substrate junction, focal adhesion, GTPase regulator activity, and cadherin binding (Fig. 3C, Table S7). These findings indicate differences in the molecular mechanisms of the new subtypes of IDH1-wtGBM.

### Associations of subtypes with the tumour immune microenvironment and programmed cell death

**Fig. 4:**
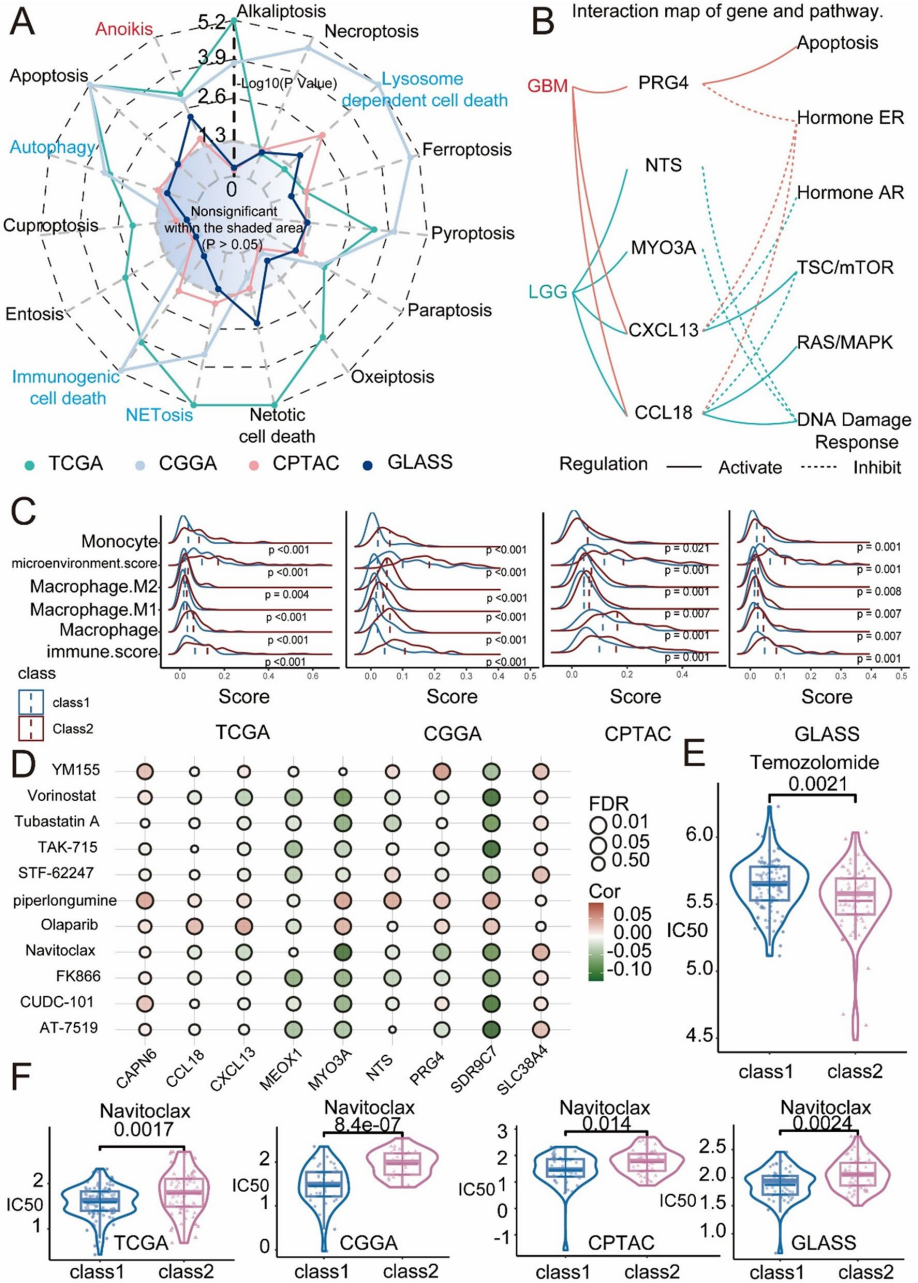
PCD and Immune Characteristics of Class 1 and Class 2 Patients, along with Potential Agents for Class 1 Patients. **A** Comparison of PCD Scores Across Molecular Subtypes in Four Databases. **B** interaction map of gene and pathway. **C** Comparison of Immune Characteristics Across Molecular Subtypes in Four Databases. **D** Bubble plot of the relationship between drugs model genes. **E** The comparison of Temozolomide’s IC50 among Molecular Subtypes. **F** Potential Agents for Class 1 in Four Databases

In the previous section of the study, we analysed the unique pathways linked to programmed cell death (PCD) and the tumour microenvironment (TME) among the new molecular subtypes of IDH1wtGBM. To confirm these distinctions, patient data from the TCGA, CGGA, GLASS, and CPTAC cohorts were used. Single-sample gene set enrichment analysis (ssGSEA) [32]was used to compute scores for PCD processes and TME-related pathways for each patient. During the examination of programmed cell death, variations were observed among each subtype of the novel IDH1wtGBM molecular subtypes, as shown in Fig. 4A and confirmed in at least one dataset. Specifically, disparities were found in lysosome-dependent cell death, NETosis, immunogenic cell death, and autophagy across the three datasets. Additionally, differences in anoikis among molecular subtypes were confirmed in all four datasets. The study revealed that class 2 PCDs were greater than class 1 PCDs. Figure 4B displays an online interaction map of the gene and pathway analysis tool GSCALite [33], which reveals that various genes in GBM and LGG activate or inhibit pathways such as the apoptosis, hormone ER, hormone AR, TSC/mTOR, RAS/MAPK, and DNA damage response pathways. It is important to note that apoptosis is a type of PCD. According to the differential analysis of immune features, class 2 scores were consistently greater than class 1 scores, as shown in Fig. 4C. Specifically, immune features such as monocytes, microenvironment scores, and macrophages were assessed. M2, Macrophage. The M1 macrophages and immune scores were consistently greater in class 2 than in class 1. Significant differences in PCD and immune characteristics were observed among the different molecular subtypes. Wu’s study [34] evaluated the status of five major types of PCD in four independent databases of 1750 glioma patients. A high ferroptosis score was closely associated with malignant progression, reduced survival rates, and a weakened antitumour immune response. Similarly, our findings revealed that individuals in class 2 with higher ferroptosis scores had poorer prognoses than those in class 1.

### Screening potential therapeutic drugs

To investigate the relationship between the model described herein and drug sensitivity, we computed the half-maximal inhibitory concentration (IC50) values for each drug in molecular subtypes of IDH1wtGBM. As shown in Fig. 4E, the IC50 of temozolomide in class 1 patients was significantly greater than that in class 2 patients. This observation is consistent with the earlier finding shown in Fig. 2F, indicating that class 2 is sensitive to temozolomide, whereas class 1 is not sensitive. We used the online drug analysis tool GSCA [23] to identify relevant drugs among the top nine differentially expressed genes between subtypes (Table S2) as a preliminary selection for potential therapeutic drugs (Fig. 4D, Table S8). Subsequently, we computed the IC50 values for the selected drugs and observed that the IC50 for Navitoclax was significantly greater in class 1 than in class 2, as shown in Fig. 4F. In a recent study, the survival rate of mice with GBM treated with the ageing drug navitoclax, an inhibitor of the antiapoptotic proteins BCL2 and BCL-xL, significantly increased compared to control mice (WT + vhc) [35]. This result suggests that patients in class 2 may be more sensitive to navitoclax, which could indicate its potential as a targeted therapy for this subgroup.

### Radiomics feature identification for molecular subtypes of IDH1-wtGBM

**Fig. 5:**
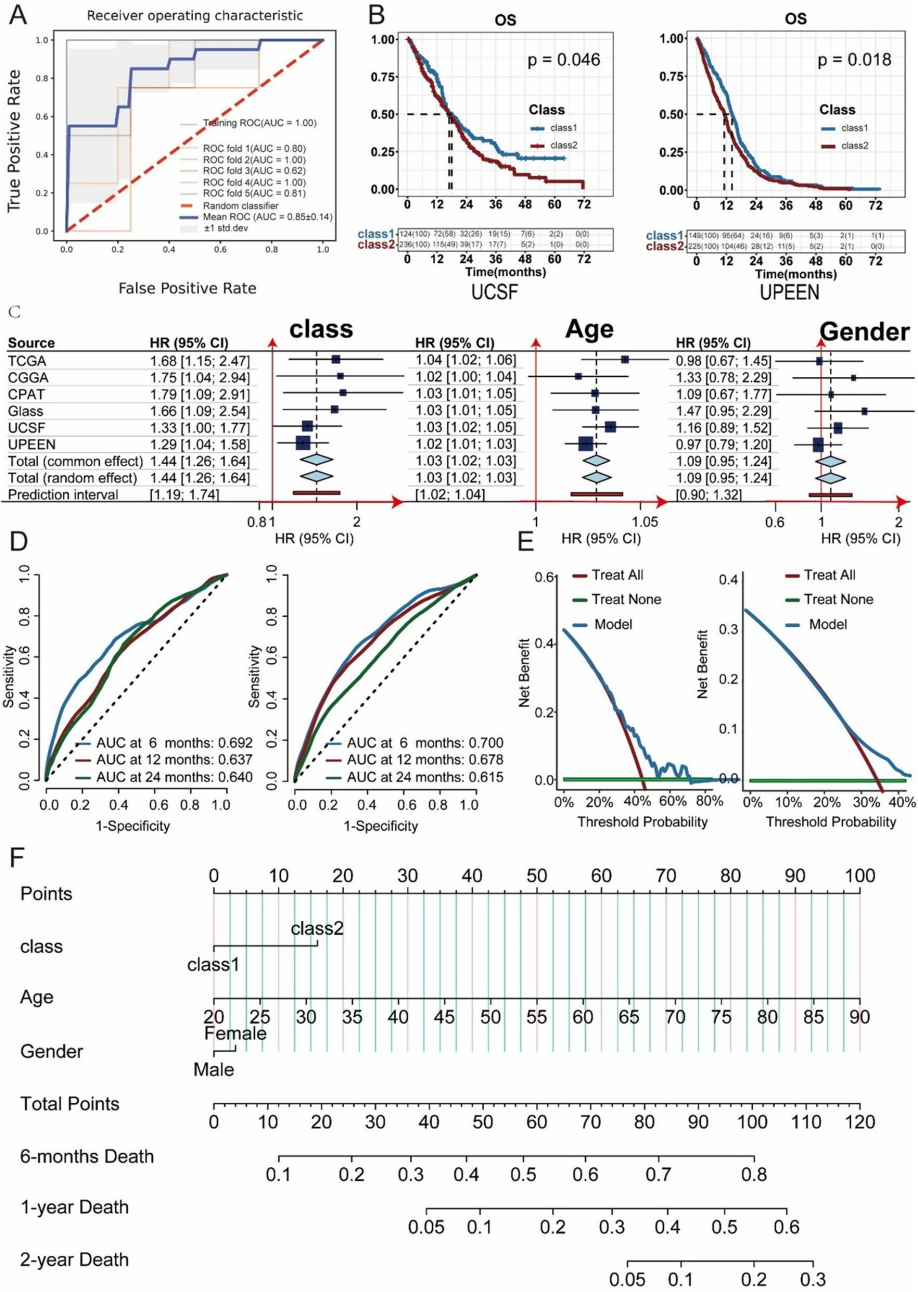
Pooling and Validation with Multiple Datasets. **A** Ten-Fold Cross-Validation of Radiomics Features for Predicting IDHwt-GBM Molecular Subtypes: Receiver Operating Characteristic (ROC) Curve. **B** Survival analysis of IDH1wtGBM patients with class 1 and class 2 in UCSF and UPEEN dataset. **C** The pooled HR of class, Age and Gender. **D** The Time-Dependent ROC and AUC of the survival model in the Training (left) and Test Sets (right). **E** The decision curve analysis for net benefit (NB) of patients avoided unnecessary interventions in the Training (left) and Test Sets (right). **F** The nomogram for model. The value of each predictor can be converted into the corresponding points according to the axis in the top of nomogram. The sum of points for each predictor can correspond to the total points axis at the bottom of the nomogram and further used to estimate the patient’s 1- and 2 years survival rate

To broaden the applicability of molecular subtyping in IDH1wtGBM patients and to provide convenience for patients ineligible for invasive biopsy and sequencing, we extracted radiomic features from four common functional magnetic resonance imaging sequences (T1, T1C, T2, and FLAIR) of 42 patients in the TCGA dataset. We built a random forest model to predict molecular subtypes and the hyperparameters of the random forest were determined by a random grid search combined with the maximum mean accuracy in fivefold cross-validation. As shown in Fig. 5A, the mean ROC curve of the model had an AUC of 0.85 ± 0.14. We then validated the predictive results of the random forest model using two additional independent cohorts with the same four common functional magnetic resonance imaging sequences. The external validation results were consistent with those from the TCGA dataset and indicated a significant survival advantage of class 1 over class 2 (Fig. 5B; UCSF, P = 0.046; UPENN, P = 0.018).

### Predictive performance and clinical net benefits of molecular subtypes of IDH1wtGBM

The meta-analysis of IDH1wtGBM across all six cohorts showed statistically significant hazard ratios for both molecular subtypes and age, as presented in Fig. 5C. The pooled hazard ratio for molecular subtypes was 1.44 (95% CI: 1.25–1.64), and for age, it was 1.03 (95% CI: 1.02–1.03). The Cox model, adjusted for molecular subtype, age, and sex, accurately predicted 6-, 12-, and 24 month survival in the TCGA training cohort. The AUC values at 6, 12, and 24 months were 0.692, 0.637, and 0.640, respectively, as shown in Fig. 5D. This predictive ability was also observed in the external test sets, where the combined AUC values were 0.700, 0.678, and 0.615 for 6-, 12-, and 24-month survival, respectively (Fig. 5D). Figure S4 shows the individual ROC curves for each external test cohort. Decision curve analysis (DCA) demonstrated that the Cox model provided greater net clinical benefit than several competing intervention strategies, such as intervention for all or intervention for none (see Fig. 5E) when 12-month survival was used as the endpoint. This illustrates the model's consistent utility and suitability for clinical implementation. To enable personalised prognostic prediction and molecular subtype screening, we constructed a nomogram based on the Cox model, as shown in Fig. 5F.

For the rapid identification of subtypes in clinical practice, our website provides models that can be used to predict IDH1wt-GBM molecular subtypes using the first nine differential genes, determine molecular subtypes based on MRI, and assess survival based on these subtypes. The website can be accessed at https://xqqcc.shinyapps.io/IDH1wtGBM/.

## Discussion

Glioblastoma (GBM) is the most lethal primary brain malignancy [36]. According to the 2021 World Health Organization classification of central nervous system tumours, GBM is divided into histological subtypes (histGBM) and molecular subtypes (molGBM) [10, 37, 38]. There is a lack of consensus in the research regarding whether the survival rates of histGBM and molGBM patients are the same. Studies led by Tesileanu et al. [12, 38, 39] indicated that survival rates are similar for histGBM and molGBM patients, while studies led by Berzero G et al. [13, 40–42] suggest otherwise. Our research, in which GBM subtypes were reclassified, yielded consistent results across multiple datasets and provided a new perspective to explain the previously conflicting findings regarding the survival rates of histGBM and molGBM patients.

Our multiomics clustering analysis revealed that in class 1, the proportions of histGBM and molGBM were similar, making it difficult to distinguish them based on molecular features. However, class 2 mainly consists of another subset of histGBM, which exhibits significantly different molecular expression than class 1. This explains the conflicting results observed in survival studies of histGBM and molGBM patients. HistGBM encompasses two distinct molecular subtypes, one that resembles molGBM and the other that differs from it. Direct comparisons between molGBM and histGBM may thus produce unstable or conflicting results.

The survival differences at the individual level reflect the molecular expression differences between class 1 and class 2. Multiple datasets, as demonstrated in Figs. 2C and 5B, consistently show this difference. Our approach differs from hypothesis-driven prognostic models, which are limited by the presence of preselected survival genes. Instead, we leveraged unbiased clustering [43], avoiding reliance on prior knowledge or assumptions about specific genes. This approach enables the identification of subtle yet significant associations between complete molecular profiles and individual outcomes, potentially revealing hidden relationships that may have been missed by traditional, survival gene-centric methods.

The clustering approach used is objective and may uncover hidden patterns. It was observed that class 2 patients were more responsive to temozolomide treatment (Fig. 2F). This difference may be attributed to the mutation patterns between class 1 and class 2. The mutation signatures enriched in class 2 exhibited the highest cosine similarity with those of SBS1, SBS6, and SBS15. SBS1 is associated with the deamination of 5-methylcytosine [28], a crucial step in DNA methylation. A recent study showed that patients with recurrent GBM after temozolomide treatment experienced a decrease in the contribution of SBS1 [29]. SBS6 and SBS15 are associated with defective DNA mismatch repair (MMR) [44] and are increased in tumours in which MMR genes are lost [45]. Temozolomide causes DNA damage in tumour cells, and defects in the MMR system cannot be efficiently repaired. This increases the likelihood of cell death, thereby increasing sensitivity to temozolomide drugs [46, 47].

Our gene set enrichment analysis (GSEA) revealed significant enrichment (FDR < 0.05) of differentially expressed genes (DEGs) in pathways such as the IL-17 signalling pathway, JAK-STAT signalling pathway, chemokine signalling pathway, PI3K-Akt signalling pathway and cytokine‒cytokine receptor interaction. The biological roles and functions of these pathways suggest that GBM class 1 and class 2 differ in terms of the tumour cell inflammatory response, immune regulation, cell proliferation and cell migration [48–50]. These differences may manifest at the individual level as differences in survival and response to treatment. The differences in sensitivity to temozolomide treatment may also be related to the enrichment of these biological pathways.

Specifically, interleukin-17 (IL-17), a proinflammatory cytokine, is upregulated in the sera and tumour tissue of GBM patients [51]. Recent research suggests that IL-17 may induce the proliferation and migration of GBM cells through activation of the PI3K/Akt1/NF-κB-p65 pathway [52]. The JAK/STAT signalling pathway facilitates the transduction of extracellular signals into intracellular physiological processes and regulates cell growth and differentiation by controlling the transcription of specific target genes [53, 54].

As shown in Fig. 3C, KEGG enrichment analysis revealed that the most significantly enriched pathways were associated with programmed cell death or the tumour immune microenvironment. Representative pathways included ferroptosis, PD-L1 expression, the PD-1 checkpoint pathway and immune-related pathways such as the B-cell receptor signalling pathway and the TNF signalling pathway. Subsequent analysis of four independent datasets confirmed significant differences in various programmed cell death patterns and the immune microenvironment between class 1 and class 2.

PCD plays a crucial role in eliminating irrelevant, infected, or potential tumour cells, underscoring its significance in maintaining homeostasis and defending against pathogens, cancer, and other pathological processes [34, 55]. During the PCD process, tumour cells release many inflammatory mediators, chemokines, and intracellular components, modifying the nearby immune microenvironment [34]. According to our analysis, patients in class 2 had significantly greater PCD scores, including scores for immunogenic cell death, ferroptosis, and apoptosis, than those in class 1.

Research suggests that blocking programmed cell death (PCD) may lead to cellular resistance to anti-PD-1/PD-L1 treatment [55]. Further investigation into the differences in PCD and the immune microenvironment between class 1 and 2 patients may provide valuable insights for improving GBM immunotherapy.

As MRI provides a readily available and comprehensive view of tumour information in the clinic, we aimed to develop a model that predicts IDHwt-GBM molecular subtypes based on MRI data. We acknowledge the inherent limitations of this approach, as the relationship between tumour MRI and genes, while they are also present [56, 57], is not fully congruent. This inevitably results in potential information loss and a possible ceiling on the model's accuracy. Despite these limitations, our predictive model has produced promising results. On two external datasets, it predicted the molecular subtypes (class 1 and class 2) of IDHwt-GBM, with the distinct survival trajectories for each subtype further validated (Fig. 5B). A meta-analysis of six additional datasets yielded consistent findings (see Fig. 5C). Building on these successes, we developed a survival model using the TCGA training set. The model integrates molecular subtypes with patient age and sex and was subsequently validated and evaluated on five external datasets. Clinicians can now easily track the survival outcomes of patients across different age-gender-subtype groups, which may inform treatment plan adjustments. The model is presented as a nomogram in Fig. 5F for easy visualisation. Interactive access is also available at https://xqqcc.shinyapps.io/IDH1wtGBM/.

Although our study revealed two GBM subtypes with distinct patient survival differences and validated the insensitivity of class 1 patients to temozolomide treatment in two datasets, certain gene mutations or enrichment patterns supported this conclusion. However, it is important to note that this study is retrospective and lacks a controlled environment for clinical experiments to account for various potential confounding factors. Therefore, we caution against directly excluding temozolomide treatment for class 1 patients, as individuals within class 1 who receive temozolomide did not show a survival disadvantage. In future clinical practice, we plan to continuously observe the survival outcomes of patients with different GBM subtypes determined based on genetic or imaging models. We will update our conclusions accordingly. Additionally, our study data come from multiple institutions, and there may be differences in detection equipment and laboratory conditions between these institutions. This is particularly true for MR images, which are very sensitive to different parameters and equipment. Although we have used standardised methods for processing, some imaging variations may still exist. Despite the incorporation of samples from multiple centres, the sample size is still limited. For knowledge discovery and model-building tasks, a larger sample size would enable increasingly accurate pattern identification. We hope studies involving larger populations will validate and extend our findings.

## Conclusion

In conclusion, we developed a precise molecular-based subtyping method for IDHwt-GBM patients by performing a multiomics analysis. This approach effectively divides IDHwt-GBM patients into two distinct groups with varying overall survival rates linked to treatment responses, copy number alterations, and PCD. Furthermore, we developed an online interactive platform capable of predicting the molecular subtypes of IDHwt-GBM in real-time, utilising both mRNA and MRI data. This convenient and timely online tool allows clinical experts to rapidly identify the molecular subtypes of patients and potential suitable therapeutic drugs. Furthermore, the platform can integrate additional clinical characteristics of patients to predict their survival status at different time points. This enables healthcare professionals to identify high-risk individuals and achieve precise diagnosis and personalised treatment. Our findings present a cost-effective solution with potential worldwide applicability within current clinical settings.

### Supplementary Information

Additional file1 (DOCX 815 KB)

## Data Availability

The supplementary document provides a description of the dataset and the URL path for accessing the relevant data.
