# Supplementary material for: Unveiling divergent treatment prognoses in IDHwt-GBM subtypes through multiomics clustering: a swift dual MRI-mRNA model for precise subtype prediction
Source: J Transl Med. 2024 Jun 18;22:578. doi: 10.1186/s12967-024-05401-6 (PMC11186189; doi:10.1186/s12967-024-05401-6)
Supplement: Supplementary file 1 — Additional file1 (DOCX 815 KB) [file 12967_2024_5401_MOESM1_ESM.docx]

**Supplementary Datasets**

**Supplementary Dataset1. TCGA**

**Supplementary Dataset2. CGGA**

**Supplementary Dataset3.GLASS**

**Supplementary Dataset4.CPTAC**

**Supplementary Dataset5.UPENN**

**Supplementary Dataset6. UCSF-PDGM**

**Supplementary Methods**

**Supplementary Method 1: Multiomics Consensus**

**Supplementary Method 2: Bioinformatics Analysis of Differences Between Subtypes of IDH1wtGBM**

**Supplementary Method 3: Radiomics Feature Extraction**

**Supplementary Method 4:** **Evaluation of multiple features**

**Dataset1. TCGA**

**Confirmation and Selection of IDH1wtGBM in the TCGA**

According to the 2021 WHO Classification of CNS Tumors (5th edition)[1], molecular pathology is the gold standard for diagnosing glioblastoma. The criteria for diagnosis include patients with IDH wild-type status and a histological diagnosis of glioblastoma (histGBM). IDH-wildtype diffuse astrocytic tumors lacking the histological features of glioblastoma are designated as molecular GBM (molGBM, WHO grade 4) if they exhibit specific molecular abnormalities, such as TERT promoter mutation, EGFR amplification, or chromosomal +7/-10 copy changes. The study followed the 2021 WHO classification standards and systematically screened and selected eligible patients from the TCGA database. In our research, we focused on exploring new subtypes of GBM using mRNA, lncRNA, and somatic mutations for multi-omics clustering. To ensure the accuracy of our results, we filtered patients who met the 2021 WHO classification criteria and possessed comprehensive mRNA, lncRNA, and somatic mutations data. The identification of 184 patients meeting the 2021 WHO classification standards was the result of a meticulous process that included comprehensive mRNA, lncRNA, and somatic mutations data.

The online tool cBioPorta (https://www.cbioportal.org/study/summary?id=lgggbm_tcga_pub) was used to extract information on TERT promoter mutation, EGFR amplification, or chromosomal +7/-10 copy changes in gliomas. The tool's original data is sourced from TCGA (https://www.cancer.gov/ccg/research/genome-sequencing/tcga), and the analytical methods and procedures adhere to the research conducted by Michele Ceccarelli and Floris P Barthel[2]. The patient information is available in Supplementary_Dataset 1.csv.

**Downloading TCGA Data**

Obtaining Clinical, treatment, mRNA, lncRNA, and Somatic Mutations Data Using the TCGAbiolinks Package [3]. Downloading MRI data was performed through TCIA (The Cancer Imaging Archive) at （<https://imaging.cancer.gov/informatics/cancer_imaging_archive.htm>）. Patients with T1, T1C, T2, and Flair sequences were selected, and matching was conducted based on patient IDs with TCGA data.

**Dataset2. CGGA**

**Confirmation and Selection of IDH1wtGBM in the CGGA**

Due to the difficulty in obtaining detailed mutation data from CGGA, according to the 2021 WHO Classification of CNS Tumors standards, we only need to exclude cases with IDH1 mutations or cases of pediatric patients from glioblastomas originally diagnosed by pathology.

**Downloading CGGA Data**

Downloading mRNA and Clinical Data from CGGA Website[4]

**Dataset3. GLASS**

**Confirmation and Selection of IDH1wtGBM in the GLASS**

The Glioma Longitudinal AnalySiS (GLASS) cohort [5]identifies individuals diagnosed with Glioblastoma (GBM) according to the guidelines outlined in the 2021 WHO Classification of CNS Tumors (5th edition). Additionally, some patients in the GLASS cohort overlap with those in the TCGA cohort. The patients from the GLASS cohort who had already been selected in the TCGA cohort based on their unique patient identifiers (ID) have been excluded.

**Downloading GLASS Data**

Obtaining clinical, treatment and mRNA data from The Glioma Longitudinal Analysis Consortium can be done through their website at <https://www.synapse.org/#!Synapse:syn17038081/wiki/585622>.

**Dataset4. CPATC**

**Confirmation and Selection of IDH1wtGBM in the CPTAC**

Due to the difficulty in obtaining detailed mutation data from Clinical Data from Clinical Proteomic Tumor Analysis Consortium (CPTAC), according to the 2021 WHO Classification of CNS Tumors standards, we only need to exclude cases with IDH1 mutations or cases of pediatric patients from glioblastomas originally diagnosed by pathology.

**Downloading CPTAC Data**

Downloading mRNA and Clinical Data from Clinical Proteomic Tumor Analysis Consortium[6]

**Dataset5 and Dataset6**

**Confirmation and Selection of IDH1wtGBM in the UPENN and** **UCSF-PDGM**

Due to the difficulty in obtaining detailed mutation data from University of Pennsylvania glioblastoma (UPENN) [7] and University of California San Francisco Preoperative Diffuse Glioma (UCSF-PDGM)[8], according to the 2021 WHO Classification of CNS Tumors standards, we only need to exclude cases with IDH1 mutations or cases of pediatric patients from glioblastomas originally diagnosed by pathology.

**Downloading UPENN and** **UCSF-PDGM**

MRI data and clinical information were obtained through TCIA (The Cancer Imaging Archive) accessible at https://imaging.cancer.gov/informatics/cancer_imaging_archive.htm. The selection process focused on patients with T1, T1C, T2, and Flair sequences to ensure a comprehensive dataset for analysis.

**Supplementary Method1 Multiomics consensus**

**Obtain elite results by reducing the dimensionality of the data.**

The Median Absolute Deviation (MAD) was used to reduce the dimensionality of the TPM-formatted datasets for mRNA and lncRNA. Genes with nonsynonymous variations, including frameshift insertions or deletions, in-frame insertions or deletions, nonsense or missense mutations, nonstop mutations, or mutations at splice sites or translation start sites were identified for the gene mutation matrix. Only genes with a mutation frequency exceeding five percent were deliberately selected.

We selected the top 500 MAD mRNA and the top 500 MAD lncRNA. Additionally, we filtered for 30 genes with a mutation frequency exceeding five percent to extract crucial data features.

**Consistent Clustering Methods for Merging Unsupervised Clustering of Multi-Omics**

This study leverages ten advanced multi-omics clustering algorithms to unveil hidden patient groupings within a cohort of 184 individuals. Employing a diverse arsenal of methods, including SNF[9], PINSPlus[10], NEMO[11], COCA[12], LRAcluster[13], ConsensusClustering[14, 15], IntNMF[16], CIMLR[17], MoCluster[18], iClusterBayes[19] , we delve into the rich tapestry of mRNA, lncRNA, and gene mutation data. To mitigate individual algorithm biases and capture a more robust landscape, we harness the power of consensus clustering. This meticulous approach integrates the insights from each method, culminating in a powerful classification scheme. To ensure the optimal number of clusters, we utilize Gaps-statistics [20] as a discerning guide. The MOVICS package [21]provides a streamlined platform for executing these multi-faceted analyses, with functions like getConsensusMOIC and getMOIC facilitating the seamless integration of clustering and consensus building.

**Supplementary Method2 Bioinformatics analysis of differences between subtypes of IDH1wt-GBM**

Differentially expressed genes (DEGs) between patient subtypes were estimated (edgeR package in R, version. 3.38.4) based on the count data of TCGA cohort, where genes with |log2FC| > 1 (FC: fold change) and P_adjust < 0.05 were selected. Gene Set Enrichment Analysis (GSEA), coupled with pathway enrichment analysis, (clusterProfiler package in R, Version 3.16.1) [22] was conducted to examine the biological functions of DEGs. GO over-representation analysis (GO-ORA) and KEGG pathway over-representation analysis (KEGG-ORA) pathway enrichment analysis were performed to exam the biological functions of significant gene (P_adjust < 0.05). Moreover, we performed protein-protein interaction (PPI) network analysis on the Differentially expressed protein using the String database (https://string-db.org/), and visualized the PPI network Cytoscape. The total mutation number and somatic copy number alteration (SCNA) of each TCGA sample were calculated (maftool package in R, Version 2.18.0) [23] on the basis of MuSe[24] preprocessed mutation data. The infiltration scores of 22 immune cells and overall immune infiltration score were estimated via R package “ConsensusTME” (version: 0.0.1.9000) [25], Single-sample gene set enrichment analysis (ssGSEA) [26] was applied to compute scores for Programmed Cell Death (PCD) processes in each patient, with reference to the patterns of PCD and key regulatory genes as outlined in the referenced articles[27, 28].Mutational signatures were assessed using the sigminer package [29] in R (Version 2.30), based on the mutational data from the TCGA cohort.

**Supplementary Method 3: Radiomics Feature Extraction**

**Image Preprocessing**

MRI data corresponding to TCGA, UPENN, and UCSF-PDGM has been stored in The Cancer Imaging Archive (TCIA, https://imaging.cancer.gov/informatics/cancer_imaging_archive.htm). Additionally, during the upload, the original authors had already performed certain standardized preprocessing [7] [8] and their processing largely adheres to the Image Biomarker Standardization Initiative[30] (IBSI). I continued to follow and adhere to the processing standards of IBSI.

**Tumor Subregion Segmentation and Unified Coding**

The segmentation and standardized encoding of tumor subregions involved datasets from TCGA, UPENN, and UCSF-PDGM. Some patients provided manually segmented tumor region masks or automatically segmented masks corrected through manual intervention. For those without provided tumor region masks, we utilized the DeepMedic[31] algorithm for automatic tumor segmentation, with subsequent manual correction to create mask files. To ensure consistent labels for different tumor subregions across these three datasets, we assigned the label 0 to the Enhancing Tumor (ET) region, 1 to the Necrotic Tumor Core (NCR), and 2 to Peritumoral edematous/infiltrated tissue (ED).

**Feature extraction**

Feature extraction was performed using the Pyradiomics package (Details of the software development company: http://pyradiomics.readthedocs.io).Radiomics features were extracted from the original image, the image after Laplacian of Gaussian filter, and the image after wavelet filter processing, utilizing the mask files of each patient.

**Feature selection**

For the TCGA dataset, we used SHAP (SHapley Additive exPlanations) values from a random forest to perform feature selection. SHAP values offer a detailed explanation of each feature's contribution to the model output, aiding in understanding the impact of each feature on the model. We excluded image features with SHAP values equal to zero and used the remaining features for model training and validation. The use of SHAP values helps identify features that are critical for the model's predictions.

**Supplementary Method 4 Evaluation of multiple features**

To assess the relationship between clinical features and overall survival, we utilized survival analysis (Cox models) from the R package. Kaplan-Meier survival curves were used to demonstrate survival differences among various groups. Prediction accuracy was demonstrated through time-dependent Receiver Operating Characteristic (ROC) curves[32], and the time-dependent Area Under the Curve (AUC) was obtained using the R package survivalROC. Decision curve analysis was performed to evaluate the net benefit (NB) of identifying true high-risk patients who should undergo intervention due to the Cox model containing subtypes of IDH1wtGBM[33]. A meta-analysis was conducted using the R package meta to combine the Hazard Ratios (HR) for IDH1wtGBM subtypes from six cohorts, utilizing both fixed-effect and random-effect models. Stratified analyses were presented through forest plots using the R package forestplot.


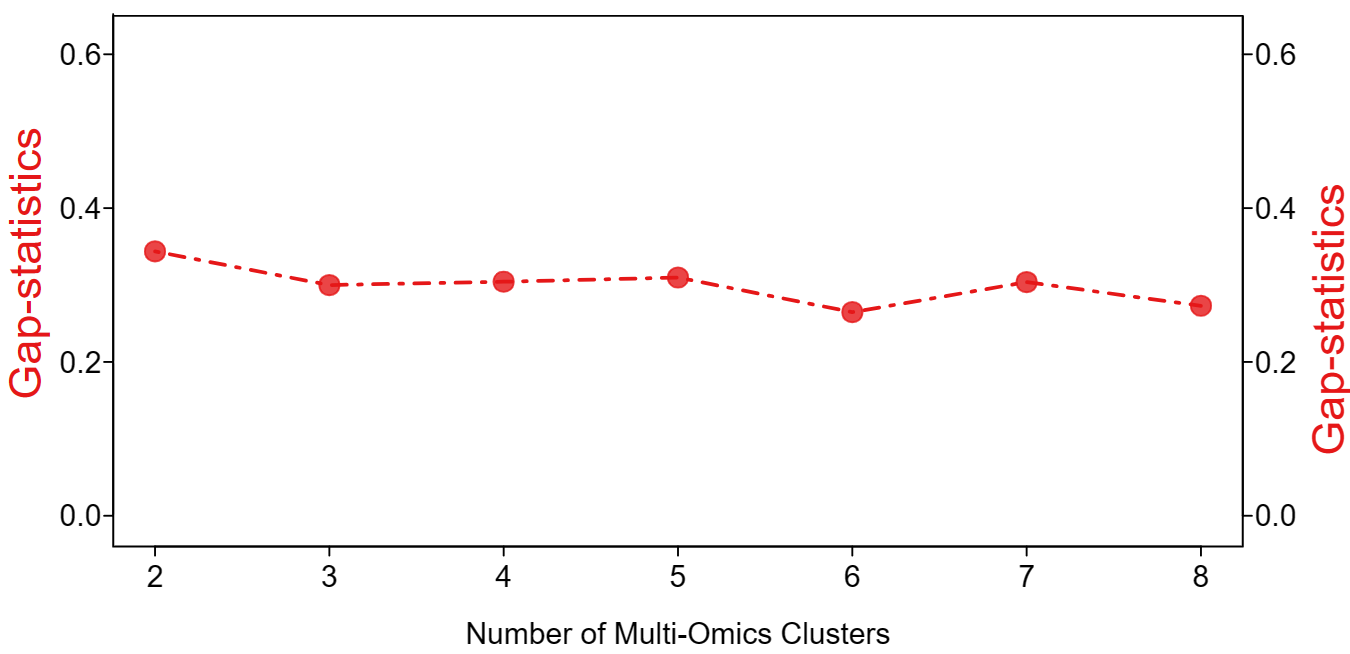


Figure S1,The Gap statistic standardizes the graph of log(Wk), where Wk is the within-cluster dispersion, by comparing it to its expectation under an appropriate null reference distribution of the data[32].


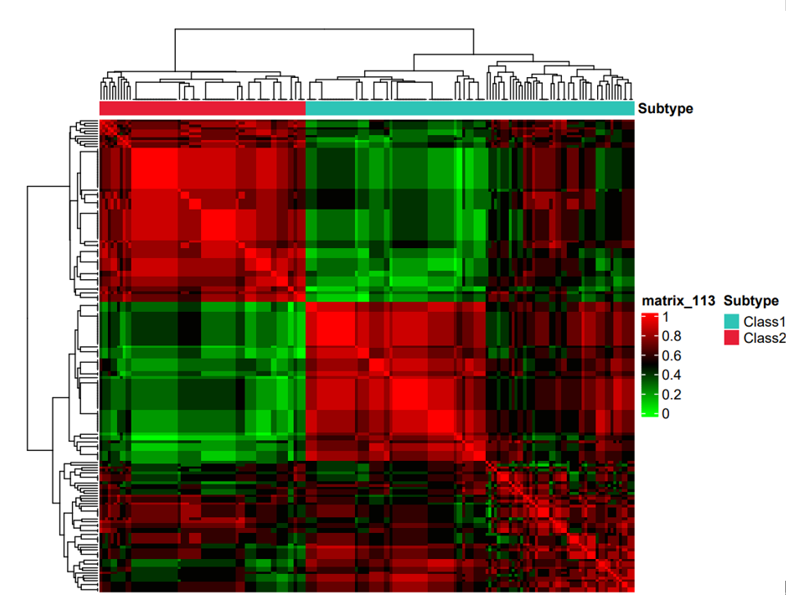


Figure S2,Consensus clustering matrix for two novel subtypes based on the 10 algorithms


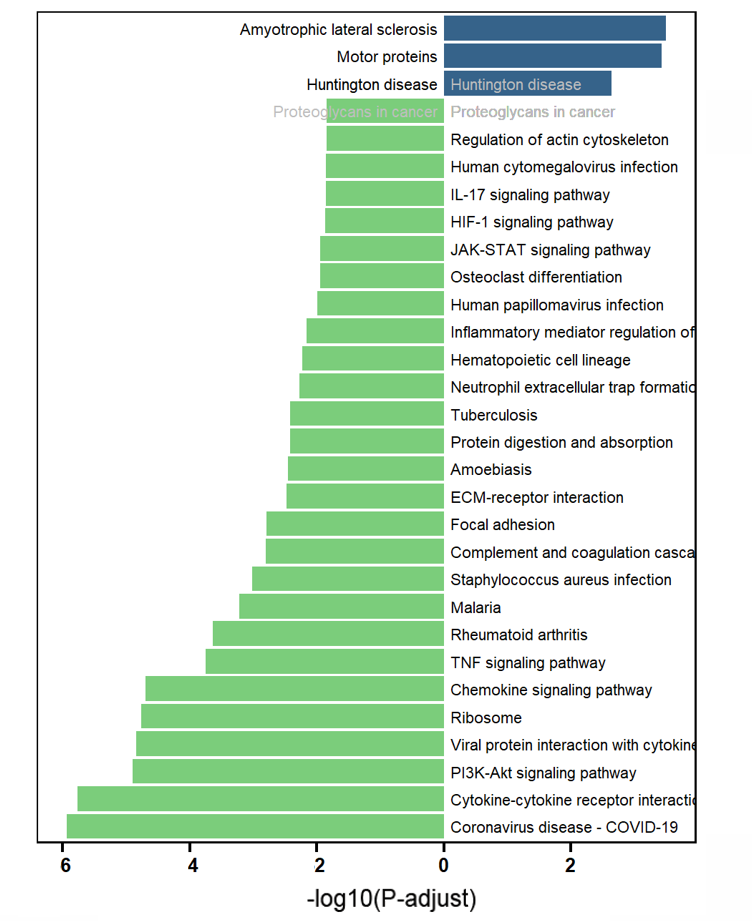


Figure S3. GSEA pathway enrichment analysis


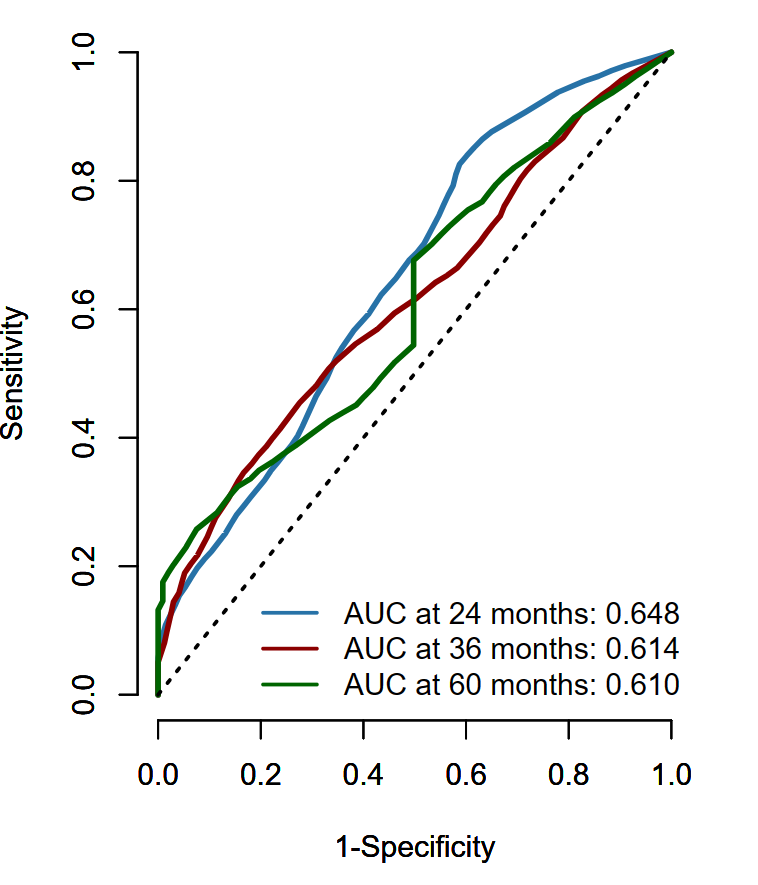

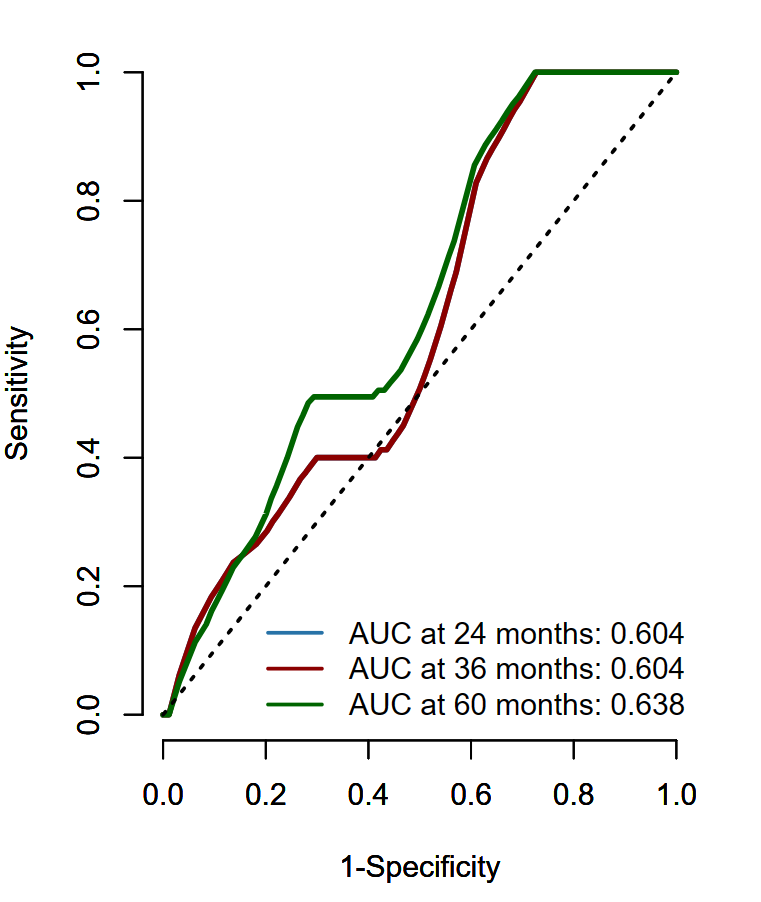


CGGA CPAT


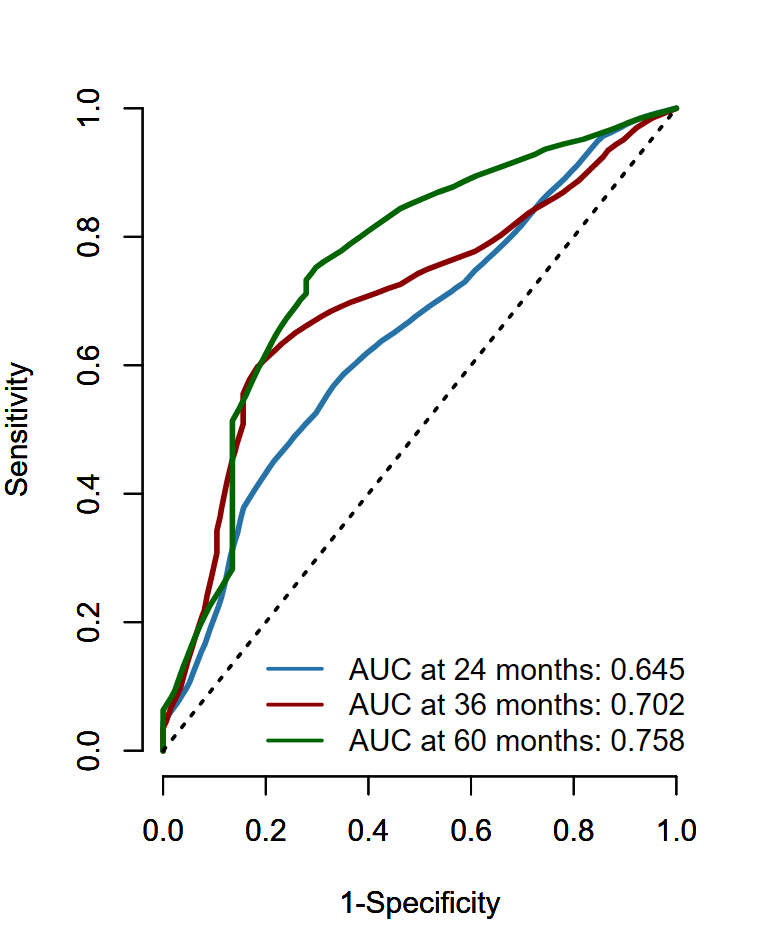

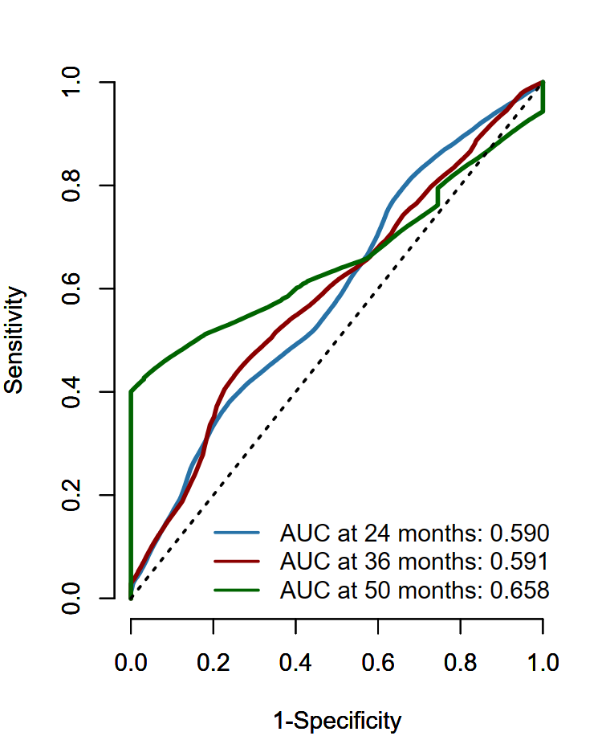


Glass Upenn


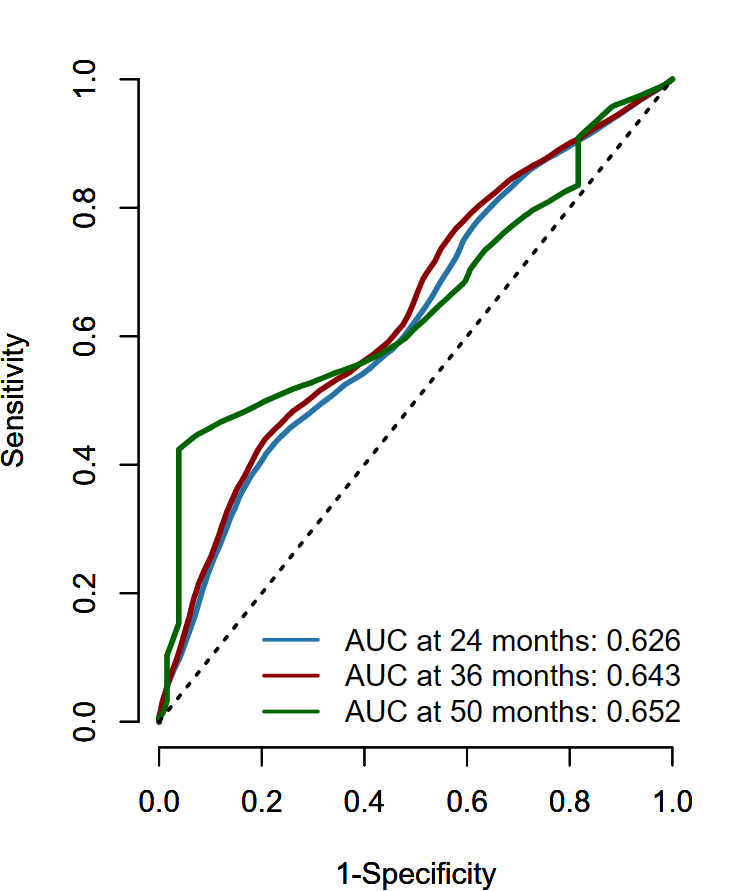


UCSF

Figure S4. ROC curves for each external test cohort.

1. Louis DN, Perry A, Wesseling P, Brat DJ, Cree IA, Figarella-Branger D, Hawkins C, Ng H, Pfister SM, Reifenberger G: **The 2021 WHO classification of tumors of the central nervous system: a summary**. *Neuro-oncology* 2021, **23**(8):1231-1251.

2. Ceccarelli M, Barthel FP, Malta TM, Sabedot TS, Salama SR, Murray BA, Morozova O, Newton Y, Radenbaugh A, Pagnotta SM *et al*: **Molecular Profiling Reveals Biologically Discrete Subsets and Pathways of Progression in Diffuse Glioma**. *Cell* 2016, **164**(3):550-563.

3. Colaprico A, Silva TC, Olsen C, Garofano L, Cava C, Garolini D, Sabedot TS, Malta TM, Pagnotta SM, Castiglioni I: **TCGAbiolinks: an R/Bioconductor package for integrative analysis of TCGA data**. *Nucleic acids research* 2016, **44**(8):e71-e71.

4. Zhao Z, Meng F, Wang W, Wang Z, Zhang C, Jiang T: **Comprehensive RNA-seq transcriptomic profiling in the malignant progression of gliomas**. *Scientific data* 2017, **4**(1):1-7.

5. Varn FS, Johnson KC, Martinek J, Huse JT, Nasrallah MP, Wesseling P, Cooper LAD, Malta TM, Wade TE, Sabedot TS *et al*: **Glioma progression is shaped by genetic evolution and microenvironment interactions**. *Cell* 2022, **185**(12):2184-2199.e2116.

6. Wang LB, Karpova A, Gritsenko MA, Kyle JE, Cao S, Li Y, Rykunov D, Colaprico A, Rothstein JH, Hong R *et al*: **Proteogenomic and metabolomic characterization of human glioblastoma**. *Cancer cell* 2021, **39**(4):509-528.e520.

7. Bakas S, Sako C, Akbari H, Bilello M, Sotiras A, Shukla G, Rudie JD, Santamaría NF, Kazerooni AF, Pati S *et al*: **The University of Pennsylvania glioblastoma (UPenn-GBM) cohort: advanced MRI, clinical, genomics, & radiomics**. *Scientific Data* 2022, **9**(1):453.

8. Calabrese E, Villanueva-Meyer JE, Rudie JD, Rauschecker AM, Baid U, Bakas S, Cha S, Mongan JT, Hess CP: **The University of California San Francisco Preoperative Diffuse Glioma MRI Dataset**. *Radiology Artificial intelligence* 2022, **4**(6):e220058.

9. Wang B, Mezlini AM, Demir F, Fiume M, Tu Z, Brudno M, Haibe-Kains B, Goldenberg A: **Similarity network fusion for aggregating data types on a genomic scale**. *Nature methods* 2014, **11**(3):333-337.

10. Nguyen H, Shrestha S, Draghici S, Nguyen T: **PINSPlus: a tool for tumor subtype discovery in integrated genomic data**. *Bioinformatics* 2019, **35**(16):2843-2846.

11. Rappoport N, Shamir R: **NEMO: cancer subtyping by integration of partial multi-omic data**. *Bioinformatics* 2019, **35**(18):3348-3356.

12. Brière G, Darbo É, Thébault P, Uricaru R: **Consensus clustering applied to multi-omics disease subtyping**. *BMC Bioinformatics* 2021, **22**(1):361.

13. Wu D, Wang D, Zhang MQ, Gu J: **Fast dimension reduction and integrative clustering of multi-omics data using low-rank approximation: application to cancer molecular classification**. *BMC genomics* 2015, **16**(1):1-10.

14. Monti S, Tamayo P, Mesirov J, Golub T: **Consensus clustering: a resampling-based method for class discovery and visualization of gene expression microarray data**. *Machine learning* 2003, **52**:91-118.

15. Wilkerson MD, Hayes DN: **ConsensusClusterPlus: a class discovery tool with confidence assessments and item tracking**. *Bioinformatics* 2010, **26**(12):1572-1573.

16. Chalise P, Fridley BL: **Integrative clustering of multi-level ‘omic data based on non-negative matrix factorization algorithm**. *PloS one* 2017, **12**(5):e0176278.

17. Ramazzotti D, Lal A, Wang B, Batzoglou S, Sidow A: **Multi-omic tumor data reveal diversity of molecular mechanisms that correlate with survival**. *Nature communications* 2018, **9**(1):4453.

18. Meng C, Helm D, Frejno M, Kuster B: **moCluster: identifying joint patterns across multiple omics data sets**. *Journal of proteome research* 2016, **15**(3):755-765.

19. Mo Q, Shen R, Guo C, Vannucci M, Chan KS, Hilsenbeck SG: **A fully Bayesian latent variable model for integrative clustering analysis of multi-type omics data**. *Biostatistics (Oxford, England)* 2018, **19**(1):71-86.

20. Tibshirani R, Walther G, Hastie T: **Estimating the Number of Clusters in a Data Set Via the Gap Statistic**. *Journal of the Royal Statistical Society Series B: Statistical Methodology* 2002, **63**(2):411-423.

21. Lu X, Meng J, Zhou Y, Jiang L, Yan F: **MOVICS: an R package for multi-omics integration and visualization in cancer subtyping**. *Bioinformatics* 2021, **36**(22-23):5539-5541.

22. Yu G, Wang LG, Han Y, He QY: **clusterProfiler: an R package for comparing biological themes among gene clusters**. *Omics : a journal of integrative biology* 2012, **16**(5):284-287.

23. Mayakonda A, Lin DC, Assenov Y, Plass C, Koeffler HP: **Maftools: efficient and comprehensive analysis of somatic variants in cancer**. *Genome research* 2018, **28**(11):1747-1756.

24. Fan Y, Xi L, Hughes DST, Zhang J, Zhang J, Futreal PA, Wheeler DA, Wang W: **MuSE: accounting for tumor heterogeneity using a sample-specific error model improves sensitivity and specificity in mutation calling from sequencing data**. *Genome Biology* 2016, **17**(1):178.

25. Jiménez-Sánchez A, Cast O, Miller ML: **Comprehensive Benchmarking and Integration of Tumor Microenvironment Cell Estimation Methods**. *Cancer research* 2019, **79**(24):6238-6246.

26. Barbie DA, Tamayo P, Boehm JS, Kim SY, Moody SE, Dunn IF, Schinzel AC, Sandy P, Meylan E, Scholl C: **Systematic RNA interference reveals that oncogenic KRAS-driven cancers require TBK1**. *Nature* 2009, **462**(7269):108-112.

27. Qin H, Abulaiti A, Maimaiti A, Abulaiti Z, Fan G, Aili Y, Ji W, Wang Z, Wang Y: **Integrated machine learning survival framework develops a prognostic model based on inter-crosstalk definition of mitochondrial function and cell death patterns in a large multicenter cohort for lower-grade glioma**. *Journal of Translational Medicine* 2023, **21**(1):588.

28. Liu T, Zhu C, Chen X, Guan G, Zou C, Shen S, Wu J, Wang Y, Lin Z, Chen L *et al*: **Ferroptosis, as the most enriched programmed cell death process in glioma, induces immunosuppression and immunotherapy resistance**. *Neuro-Oncology* 2022, **24**(7):1113-1125.

29. Wang S, Li H, Song M, Tao Z, Wu T, He Z, Zhao X, Wu K, Liu X-S: **Copy number signature analysis tool and its application in prostate cancer reveals distinct mutational processes and clinical outcomes**. *PLOS Genetics* 2021, **17**(5):e1009557.

30. Zwanenburg A, Vallières M, Abdalah MA, Aerts H, Andrearczyk V, Apte A, Ashrafinia S, Bakas S, Beukinga RJ, Boellaard R *et al*: **The Image Biomarker Standardization Initiative: Standardized Quantitative Radiomics for High-Throughput Image-based Phenotyping**. *Radiology* 2020, **295**(2):328-338.

31. Kamnitsas K, Ledig C, Newcombe VF, Simpson JP, Kane AD, Menon DK, Rueckert D, Glocker B: **Efficient multi-scale 3D CNN with fully connected CRF for accurate brain lesion segmentation**. *Medical image analysis* 2017, **36**:61-78.

32. Hastie T, Tibshirani R, Walther G: **Estimating the number of data clusters via the Gap statistic**. *J Roy Stat Soc B* 2001, **63**:411-423.
